# Ameliorative potential of desalted *Salicornia europaea L*. extract in multifaceted Alzheimer’s-like scopolamine-induced amnesic mice model

**DOI:** 10.1038/s41598-018-25381-0

**Published:** 2018-05-08

**Authors:** Govindarajan Karthivashan, Shin-Young Park, Mee-Hyang Kweon, Joonsoo Kim, Md. Ezazul Haque, Duk-Yeon Cho, In-Su Kim, Eun-Ah Cho, Palanivel Ganesan, Dong-Kug Choi

**Affiliations:** 1 Department of Biotechnology, College of Biomedical and Health Science, Research Institute of Inflammatory Diseases Konkuk University, Chungju, 27478 Republic of Korea; 2 Department of Applied Life Science, Graduate school of Konkuk University, Chungju, 27478 Republic of Korea; 3 Nanotechnology research center College of Biomedical and Health Science, Konkuk University, Chungju, 27478 Republic of Korea; 4 Research center, Phyto corporation, Seoul, 08826 Republic of Korea

## Abstract

The *Salicornia europaea L*. (SE) plant is a halophyte that has been widely consumed as a seasoned vegetable, and it has been recently reported to counteract chronic diseases related to oxidative and inflammatory stress. In this study, we performed an initial phytochemical analysis with *in vitro* biochemical tests and chromatographic profiling of desalted and enzyme-digested SE ethanol extract (SE-EE). Subsequently, we evaluated the anti-neuroinflammatory and ameliorative potential of SE-EE in LPS-inflicted BV-2 microglial cells and scopolamine-induced amnesic C57/BL6N mice, respectively. SE-EE possess considerable polyphenols and flavonoids that are supposedly responsible to improve its bio-efficacy. SE-EE dose-dependently attenuated LPS-induced inflammation in BV-2 cells, significantly repressed behavioural/cognitive impairment, dose-dependently regulated the cholinergic function, suppressed oxidative stress markers, regulated inflammatory cytokines/associated proteins expression and effectively ameliorated *p*-CREB/BDNF levels, neurogenesis (DCX stain), neuron proliferation (Ki67 stain) in scopolamine-administered mice. Thus, SE-EE extract shows promising multifactorial disease modifying activities and can be further developed as an effective functional food, drug candidate, or supplemental therapy to treat neuroinflammatory mediated disorders.

## Introduction

Neurodegenerative disease are extensive clinical complications associated with progressive dysfunction and structural abrogation of functional neurons that result in neuronal cell death^1^. Alzheimer’s disease (AD) is one of the most commonly reported age-related neurodegenerative diseases. Epidemiologic data shows that among the world’s population, around 33.9 million people have been diagnosed with AD, and this figure is expected to triple over the next 40 years^2^. AD is characterized by impairments in hippocampal neurogenesis, associated cognitive dysfunction, and memory deficits^3,4^. Although the etiology of AD is still being explored, its associated pathophysiological events – such as cholinergic system dysfunction, β-amyloid (Aβ) deposits, oxidative stress, and inflammation – have been consistently reported to play influential roles in the induction and progression of the AD pathology^5,6^. A typical cholinergic system in the brain convincingly influence hippocampal neurogenesis and cognitive function by modulating brain-derived neurotrophic factor (BDNF) and cAMP response element-binding protein (CREB) mediated neurogenic mechanisms^7,8^. Subsequently, newly-formed neural progenitor cells at the dentate gyrus of the hippocampus develop as new functional neurons and integrate with the existing neuronal circuit, thereby playing a pivotal role in cognition and memory^9^.

In the event of AD, learning and memory impairments have been primarily induced by cholinergic dysfunction, such as elevated acetylcholinesterase (AChE) activity and relative inhibition of acetylcholine (ACh) release in the central nervous system (CNS)^10^. Thus, one of the most effective clinical treatments for AD consists of cholinergic revival therapy provided by AChE inhibitors such as donepezil, galantamine, etc., but it is found to provide symptomatic relief^11^. Regarding brain tissue, the hippocampus and amygdala regions are highly susceptible to oxidative stress due to their high oxygen requirements, and these are reported to impair synaptic plasticity in ageing AD disorder^12,13^. The neuronal inflammatory cascade, apparently precluded by the cholinergic malfunction, oxidative stress milieu and neuronal cell death, was also reported to play a major role in AD pathology^14^. From the above, identifying potential candidates and targeting these multifactorial pathological events associated with AD progression is a promising therapeutic strategy to treat AD. Accordingly, the search for safe, effective drug candidates focusing on these multiple targets is still in progress.

*Salicornia europaea L*. (SE), commonly known as glasswort, is a halophytic plant that belongs to the Amaranthaceae family and is native to the Mediterranean, but it is also found abundantly in coastal regions of East Asia. It is also known as *Salicornia herbacae L*, and is vernacularly named as ‘hamcho’ in Korea^15^. As the plant habitually grows in high-salt coastal marshes, it was reported to possess abundant bioactive defence secondary metabolites, which aids the plant to overcome profuse salt stress. It is commonly consumed, as a raw vegetable or as a nutritious fermented food in Korea and European countries^16,17^. SE has recently emerged as a commercial, edible halophyte, and it can be cultivated using sea water irrigation without fertilizers or pesticides^18^. Phytochemical investigations of SE have reported the presence of carbohydrates, proteins, minerals, oils, phenolic compounds, flavonoids, sterols, saponins, alkaloids, and tannins^16,19^. In addition, accumulating evidence suggests that SE has therapeutic potential as an anti-microbial, anti-oxidative, anti-tumor, anti-adipogenic, anti-vascular neointima, anti-hyperlipidemic and anti-diabetic agent^20–22^. Although numerous studies have documented the therapeutic potential of SE for various diseases, to the best of our knowledge, we are the first to investigate the anti-neuroinflammatory and anti-amnesic effects of desalted and enzyme-digested SE ethanol extract (SE-EE). This study examined its applicability using LPS induced BV-2 microglial cells *in vitro*, and scopolamine-induced amnesic mice *in vivo*.

## Results

### Chromatographic and Phytochemical profiling of SE-EE

**Figure 1:**
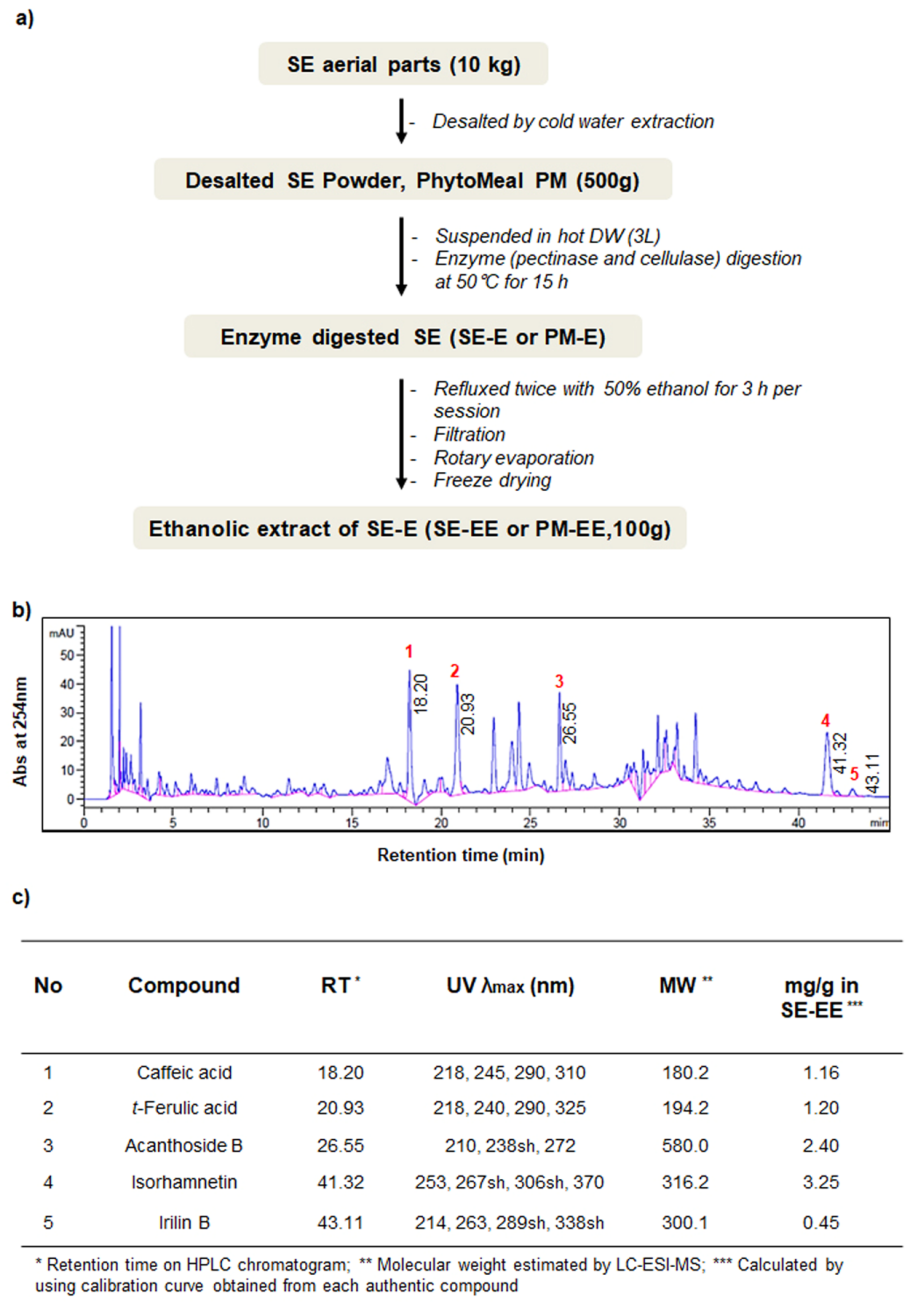
SE-EE preparation and chromatographic profile: (**a**) Schematic representation of the SE-EE preparation procedure; (**b**) representative HPLC chromatogram of SE-EE acquired from the desalted *Salicornia europaea*; (**c**) Tabulation of the identified compounds from the chromatographic profile of SE-EE.

**Figure 2:**
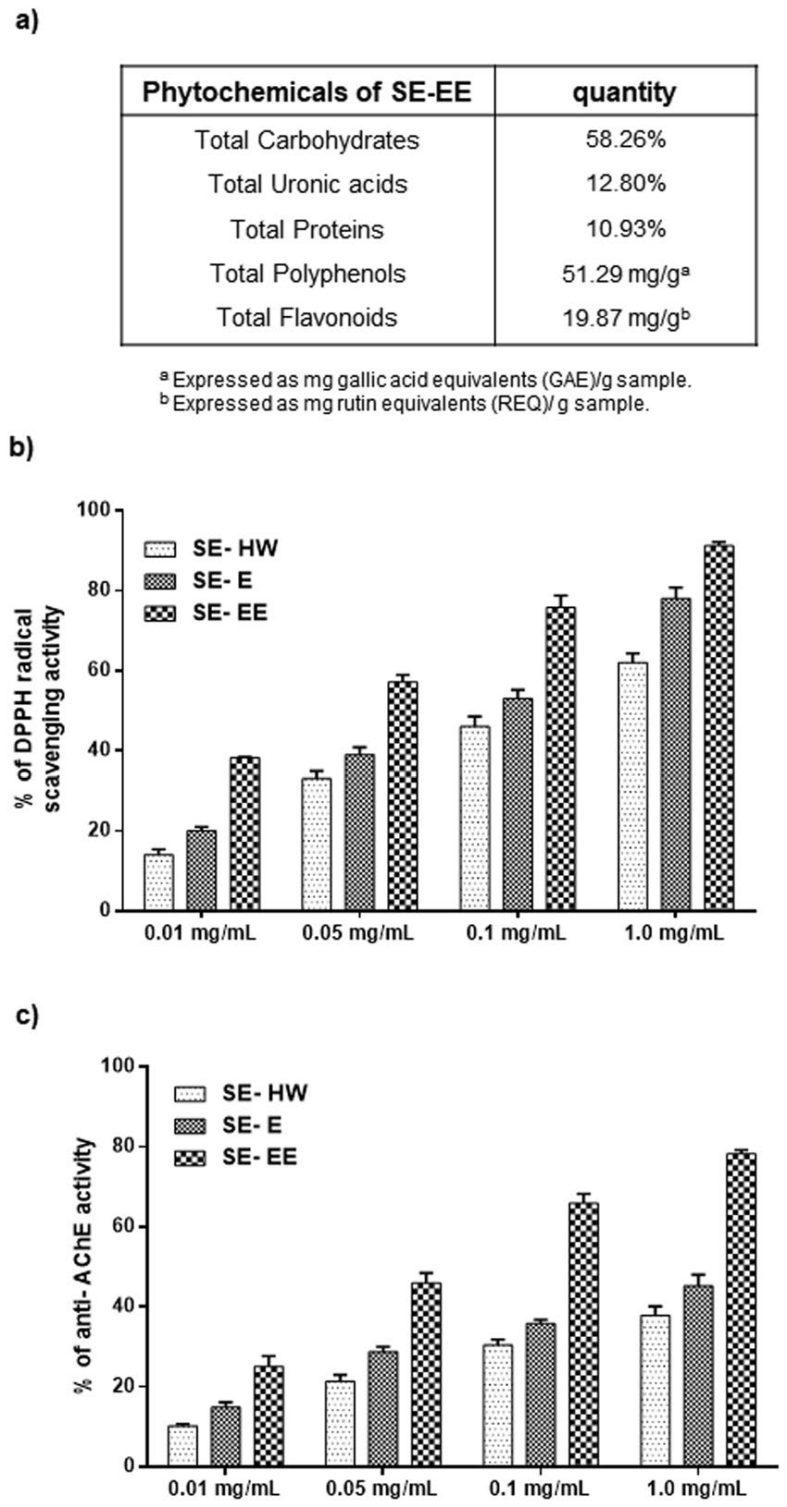
Phyochemical investigations and *in vitro* free-radical scavenging/anti-AChE activities of SE-EE: (**a**) Profiling and quantification of SE-EE phytochemicals; (**b**) *in vitro* DPPH free radical scavenging activity and (**c**) *in vitro* anti-AChE potential of enzyme-digested desalted SE-EE, compared to hot water SE (SE-HW) extracts and conservative SE ethanol (SE-E) extracts at varying concentrations.

Based on the obtained SE-EE chromatogram, retention time and, mass spectra, five major compounds were identified including caffeic acid, trans-ferulic acid, acanthoside B, isorhamnetin, irilin B (Fig. 1b). Each compound was subsequently quantified (Fig. 1c) using commercial authentic standards with their corresponding standard curves. The phytochemical profiling of SE-EE revealed 58.26%, 12.80% and 10.93% in total carbohydrates, total uronic acids and total proteins, respectively. The total phenolic and flavonoid contents of SE-EE were measured as 51.29 mg gallic acid equivalent/g sample and 19.87 mg rutin equivalent/g sample, respectively (Fig. 2a).

### *In vitro* anti-oxidant and anti-AChE activities of SE-EE

As shown in Fig. 2b and c, all the various methodologic extracts of SE dose-dependently exhibit substantial anti-oxidant and anti-AChE activity. Among them, enzyme digested SE-EE exhibited a higher anti-oxidant and anti-AChE activity with the lowest IC_50_ values of 0.47 ± 0.08 mg/mL and 0.92 ± 0.10 µg/mL, respectively, followed by ethanolic extract (SE-E) > hot water extract (SE-HW). Thus, we pursued enzyme digested SE-EE for further disease modifying investigations in an amnesic AD-like model.

### SE-EE attenuated LPS induced neuro-inflammation in BV-2 microglial cells

#### Effects on cell viability and NO release

**Figure 3:**
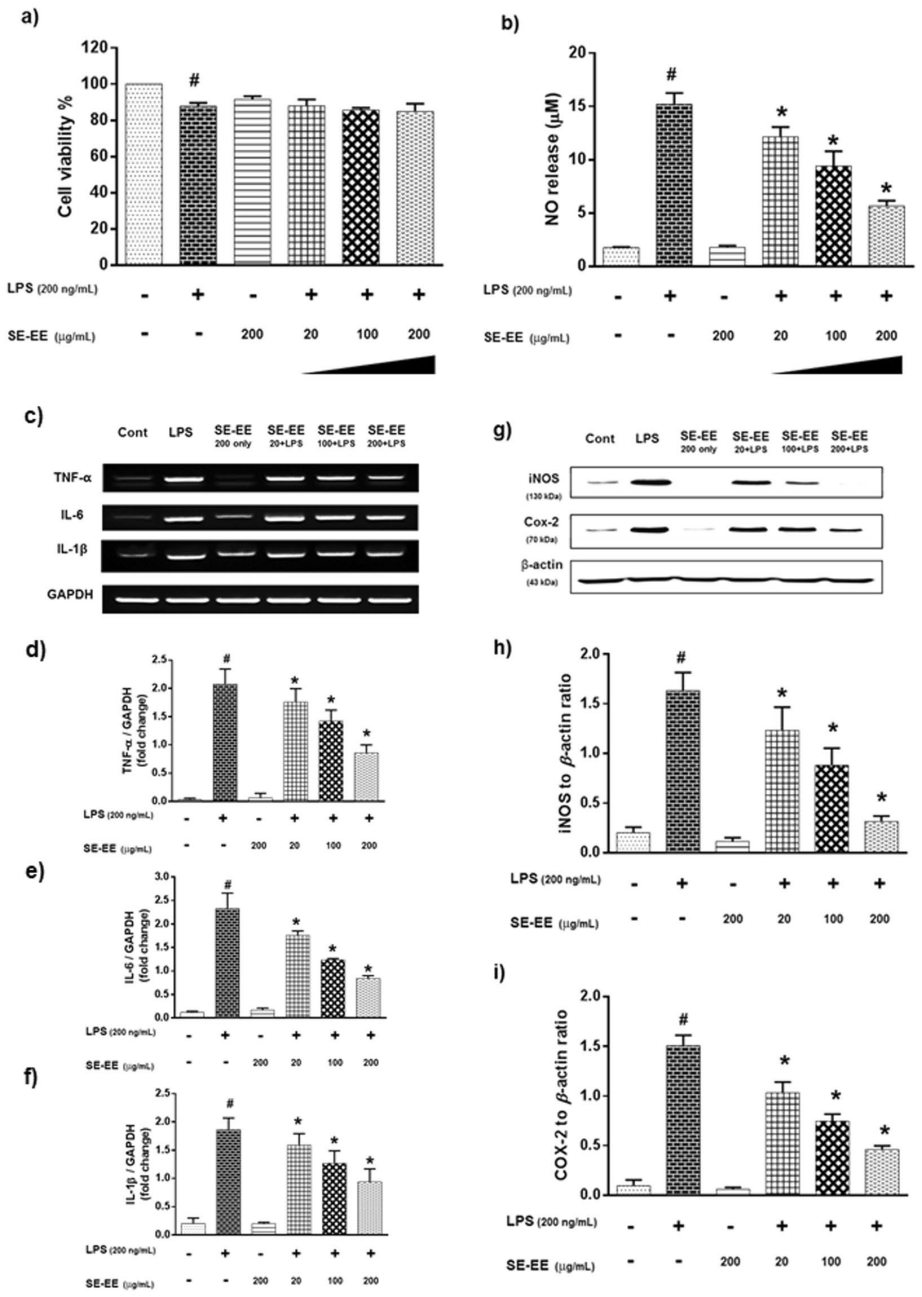
Effects of SE-EE on LPS induced neuro-inflammation in BV-2 microglial cells: BV-2 cells were incubated with the described concentrations of SE-EE for 30 min before LPS induction and incubated for 6 h (reverse transcription-polymerase chain reaction analysis) and 24 h (NO release and western blot analysis). (**a**) Cell viability percentage and (**b**) NO release in BV-2 microglial cells; Total RNA was prepared for RT-PCR analysis (**c**) TNF-α, IL-1β and IL-6 gene expression and (**d**–**f**) semi-quantification of respective gene expression relative to GAPDH was achieved using the ImageJ software; Cell lysates were electrophoresed, (**g**) iNOS and COX-2 protein expressions were determined via Western blotting (respective markers were acquired by cropping from the same gel, full length blots are in Supplementary data- S.3.1) and (**h** and **i**) quantification of inflammatory protein expression relative to β-actin was achieved using the ImageJ software. Data are expressed as mean ± SD of three independent experiments. One-way ANOVA-Tukey’s multiple comparison test was performed. ^#^*p* < 0.05 LPS treated cells compared with the control cells; *p < 0.05 other sample concentrations compared with LPS treated cells.

To determine the cytotoxicity profile of SE-EE, we exposed BV-2 microglial cells to varied concentrations (20, 100 and 200 µg/mL) of SE-EE and scrutinized the toxic profile based on the glial cell viability. After 24 h of cell incubation with different concentrations of SE-EE in the presence or absence of LPS (200 ng/mL), the viability of the cells was measured via an MTT assay. Treatment with SE-EE alone at a higher concentration of 200 µg/mL did not exhibit a significant cell toxicity. The SE-EE and LPS treated cells show a reduction in the cell viability percentage, and almost all evaluated SE-EE concentrations with LPS stimulation exhibited more than 80% cell viability percentage, indicating a non-toxic potential (Fig. 3a). Subsequently, to evaluate the effects of SE-EE on NO production, microglial cells were pre-treated with indicated concentrations (20, 100 and 200 µg/mL) of SE-EE for 1 h, followed by LPS (200 ng/mL) stimulation. After 24 h, the culture medium of the cells was harvested and subjected to an NO assay. From Fig. 3b, the LPS stimulation significantly (*p* < 0.05) increased the NO release compared to the control group. Pre-treatment of SE-EE dose-dependently (*p* < 0.05) suppressed NO release in LPS stimulated BV-2 microglial cells.

#### Effects on proinflammatory cytokine levels

Proinflammatory cytokines (TNF-α, IL-1β, IL-6) play a major role in the progression and expansion of neuro-inflammation. Accordingly, we investigated the effects of SE-EE on proinflammatory cytokine levels of LPS stimulated BV-2 microglial cells. BV-2 microglial cells were stimulated with LPS (200 ng/mL) in the presence or absence of SE-EE at indicated concentrations (20, 100 and 200 µg/mL). The RT-PCR data for 6 h LPS stimulation in BV-2 microglial cells exhibited a significant (*p* < 0.05) increase in the mRNA levels of TNF-α, IL-1β, IL-6 with approximately a two-fold increase compared to control cells (Fig. 3c–f). Pre-treatment of SE-EE, 1 h before LPS stimulation, significantly subsided the mRNA levels of evaluated proinflammatory cytokines. Precisely, at the highest evaluated dose of 200 µg/mL, SE-EE decreased the mRNA levels of TNF-α, IL-1β, IL-6 by nearly one-fold less than the LPS-stimulated cells.

#### Effects on iNOS and COX-2 protein expression

Inducible nitric oxide synthase (iNOS) and cyclooxygenase-2 (COX-2) have been extensively investigated as inflammatory mediators and were reported to be stimulated or co-stimulated by proinflammatory cytokines, thereby playing a pivotal role in the inflammatory cascade. In this regard, we further investigated the effects of SE-EE on iNOS and COX-2 level expressions in LPS-stimulated BV-2 microglial cells. BV-2 microglial cells were stimulated with LPS (200 ng/mL) in the presence or absence of SE-EE at the indicated concentrations (20, 100 and 200 µg/mL). The Western blot data of LPS stimulation on BV-2 microglial cells exhibited a significant (*p* < 0.05) increase in the protein expression of iNOS and COX-2, greater than one-fold, compare to control cells (Fig. 3g–i). Pre-exposure of SE-EE, 1 h before LPS stimulation, significantly (*p* < 0.05) attenuated LPS induced iNOS and COX-2 protein expression. Precisely at a dose of 200 µg/mL, SE-EE reduced the protein levels of iNOS and COX-2 by almost one-fold less than the LPS-stimulated cells. This is in accordance with the reduction in the mRNA levels of the pro-inflammatory cytokines. Together these data elucidate the anti-neuroinflammatory potential of SE-EE extracts by constraining the proinflammatory cytokine-associated regulation of inflammatory mediators in glial cell-mediated neuroinflammation.

### SE-EE ameliorated scopolamine inflicted cognitive and behavioral impairment in C57/BL6N mice

#### Effects on anti-amnesic learning and memory retention

**Figure 4:**
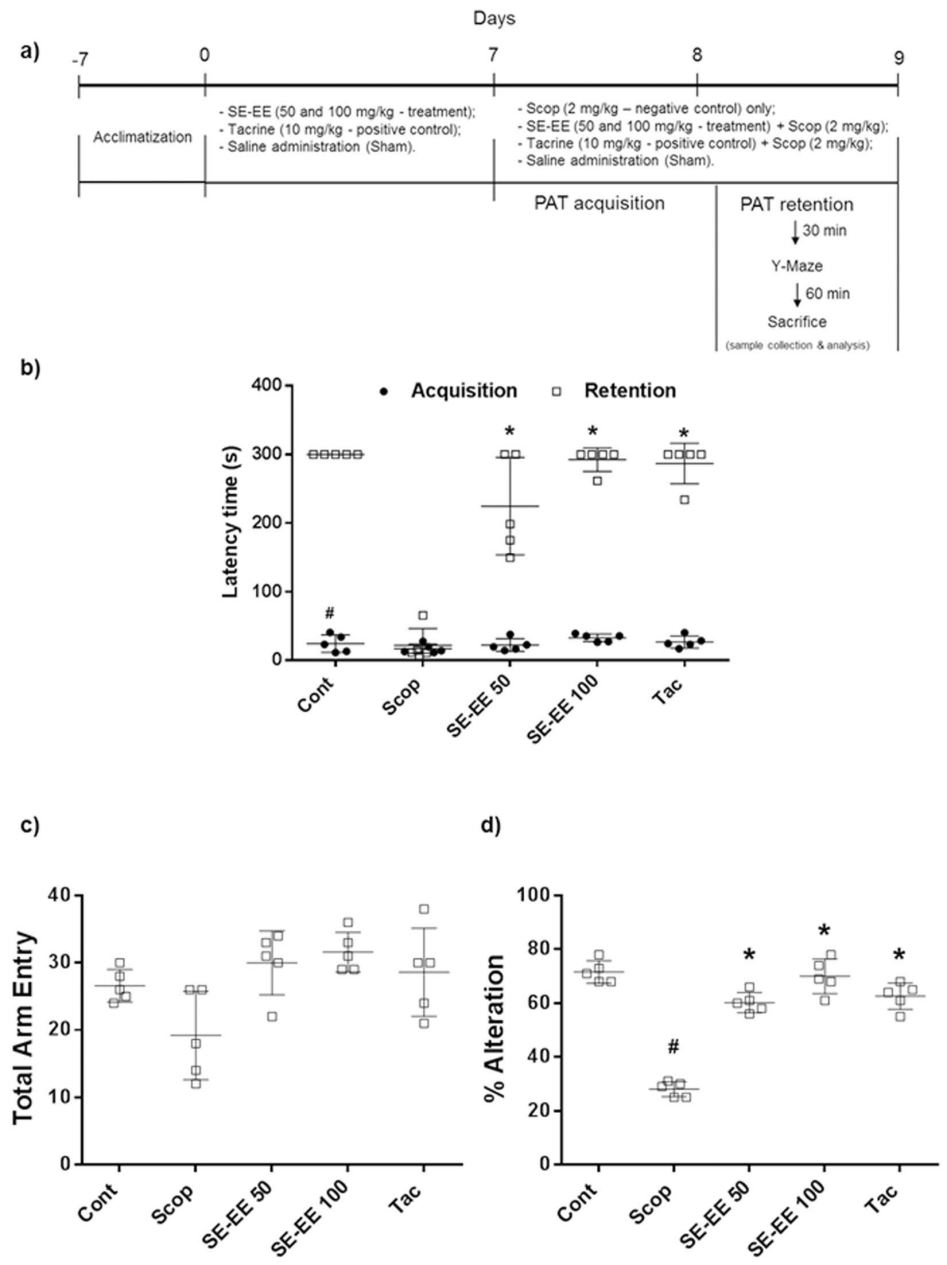
Effects of SE-EE on scopolamine inflicted cognitive and behavioral impairment in C57/BL6N mice: (**a**) The design of the experiments with animals; (**b**) In the step-through passive avoidance test, the latency variance in the acquisition and retention phase was acquired; (**c**) In the spontaneous alternation performance (Y-maze test), the total arm entry and percentage of alternation was acquired. Data are expressed as mean ± SD (n = 6). One-way ANOVA-Tukey’s multiple comparison test was performed. ^#^*p* < 0.05 compared with the control group; *p < 0.05 other treated groups compared with the scopolamine group.

The learning and memory-retentive potential of treated animals in a specific environmental condition was investigated by evaluating the time latencies of animals recorded using a step-through passive avoidance test. From Fig. 4b, during the acquisition session, all groups exhibited a non-significant pattern in their step-through latency times. Subsequently, during the retention phase, scopolamine administration significantly reduced the latency time to 22 ± 25 s, compared to the control group of 300 s. Pre-treatment of SE-EE dose dependently ameliorated the memory retention, with a significant (*p* < 0.05) improvement in the latency times, compared to the scopolamine-treated group. At the indicated higher dose of 100 mg/kg of b.w, SE-EE adequately reinstated the latency time to 292 ± 17 s nearby the positive control, the tacrine (10 mg/kg of b.w) group, at 287 ± 29 s.

#### Effects on anti-amnesic cognitive and exploratory behavior

The impulsive memory functioning and probing behavior of the animals was investigated by recording the spontaneous alternation behavior using the Y-maze test. In this study, the spontaneous alternation percentage for the scopolamine (2 mg/kg of b.w) treated group was significantly (*p* < 0.05) reduced with a value of 28 ± 2.83%, compared to the control group, at 71.6 ± 4.16% (Fig. 4d). SE-EE treated animals exhibited a significant (*p* < 0.05) increase in the spontaneous alternation percentage compared to the scopolamine-treated group, dose-dependently. Interestingly, at the evaluated higher dose, SE-EE (100 mg/kg of b.w) elevated the spontaneous alternation percentage with a value of 70 ± 6.44%, which was relatively higher than that for the tacrine treated group of 62.6 ± 4.96% and proximal to the control group. In terms of the total arm entries (Fig. 4c), no significant differences were observed among the treated groups.

### SE-EE attenuated scopolamine inflicted cholinergic impairment, lipid peroxidation and oxidative stress in C57/BL6N mice

#### Effects on AChE activity in hippocampus and cerebral cortex

**Figure 5:**
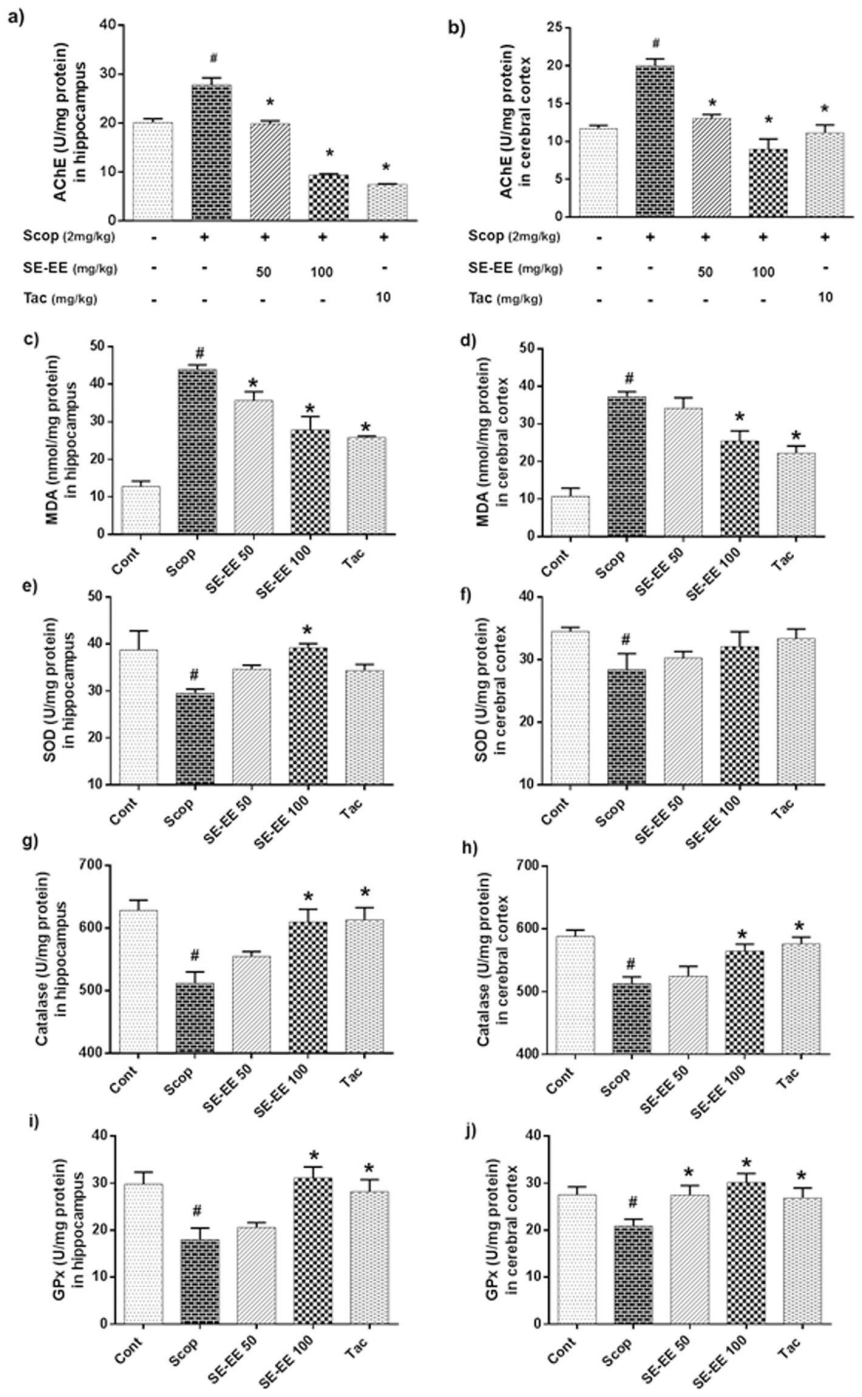
Effects of SE-EE on scopolamine-inflicted cholinergic impairment, lipid peroxidation and oxidative stress in C57/BL6N mice: AChE activity – (**a**) AChE levels in hippocampus, (**b**) AChE levels in cerebral cortex. Tacrine (10 mg/kg) was used as positive control; Endogenous level of oxidative stress/antioxidant biomarkers – (**c**,**e**,**g** and **i**) represents the lipid peroxidation (MDA), Superoxide dismutase (SOD), Catalase (CAT) and Glutathione peroxidase (GPx) levels in the hippocampus respectively; (**d**,**f**,**h** and **j**) represents the MDA, SOD, CAT and GPx levels in the cerebral cortex of the treated animals, respectively. The data are expressed as mean ± SD (n = 5, pooled biological replicates). One-way ANOVA-Tukey’s multiple comparison test was performed. ^#^*p* < 0.05 compared with the control group; *p < 0.05 other treated groups compared with the scopolamine group.

Acetylcholinesterase (AChE) enzyme plays major role in the breakdown of acetylcholine, a pivotal neurotransmitter in cholinergic system. From Fig. 5a and b, scopolamine-administered mice significantly increased AChE activity in both the hippocampus and cerebral cortex with values of 27.78 ± 1.46 U/mg protein and 20.02 ± 0.88 U/mg protein respectively, compared to control group with the values of 20.13 ± 0.75 U/mg protein and 11.70 ± 0.42 U/mg protein respectively. SE-EE treated animals exhibited a significant (*p* < 0.05) decrease in AChE activity in both hippocampus and cerebral cortex compared to the scopolamine treated group, dose-dependently. SE-EE (100 mg/kg of b.w) exhibited a significantly (*p* < 0.05) higher suppression of AChE activity in both hippocampus and cerebral cortex with values of 9.35 ± 0.25 U/mg protein and 8.96 ± 1.37 U/mg protein, respectively, and this can be correlated with the tacrine-treated groups with values of 7.40 ± 0.19 U/mg protein and 11.14 ± 1.05 U/mg protein, respectively.

#### Effects on lipid peroxidation and antioxidant biomarkers in hippocampus and cerebral cortex

Scopolamine-induced amnesia is strongly reported to be connected with increased oxidative stress in the brain. Accordingly, we evaluated the lipid peroxidation by malonaldehyde (MDA) and also measured the antioxidant biomarker (SOD, CAT and GPx) levels in both the hippocampus and the cerebral cortex. From the Fig. 5c and d, it was clear that the scopolamine-administered mice exhibited significantly (*p* < 0.05) elevated lipid peroxidation activity in both the hippocampus and cerebral cortex with values of 43.95 ± 1.18 nmol/mg protein and 37.25 ± 1.36 nmol/mg protein, compared to the control (12.76 ± 1.44 nmol/mg protein and 10.71 ± 2.20 nmol/mg protein) groups. Subsequently, Fig. 5e–j show that the scopolamine-administered mice exhibited significantly (*p* < 0.05) suppressed antioxidant enzymes SOD, CAT, GPx activity in both the hippocampus and cerebral cortex with values of 29.52 ± 0.86, 511.94 ± 17.94, 17.99 ± 2.48 U/mg protein and 28.39 ± 2.57, 512.50 ± 11.08, 20.90 ± 1.46 U/mg protein compared to the control groups (38.76 ± 4.05, 628.30 ± 16.36, 29.78 ± 2.58 U/mg protein and 34.50 ± 0.65, 588.00 ± 10.40, 27.54 ± 1.69 U/mg protein). Alternatively, pre-treatment of SE-EE significantly (*p* < 0.05) inhibited scopolamine-induced amnesic oxidative stress milieu by suppressing the lipid peroxidation activity and enhancing the antioxidant enzyme activities. Precisely, at a higher dose, SE-EE (100 mg/kg of b.w.) suppressed the level of lipid peroxidation activity in both the hippocampus and cerebral cortex with values of 27.80 ± 3.61 nmol/mg protein and 25.44 ± 2.73 nmol/mg protein and also elevated the antioxidant enzymes SOD, CAT, GPx activities with values of 39.19 ± 0.89, 609.57 ± 20.46, 31.12 ± 2.33 U/mg protein and 32.08 ± 2.37, 564.73 ± 11.17, 30.15 ± 1.90 U/mg protein respectively. This was proximal to the tacrine treated (positive control) group which suppressed the level of lipid peroxidation activity in both hippocampus and cerebral cortex with values of 25.75 ± 0.47 nmol/mg protein and 22.21 ± 1.93 nmol/mg protein and also elevated the antioxidant enzymes SOD, CAT, GPx activity with values of 34.35 ± 1.30, 612.98 ± 19.59, 28.18 ± 2.58 U/mg protein and 33.36 ± 1.54, 576.08 ± 10.95, 26.86 ± 2.13 U/mg protein, respectively.

### SE-EE attenuated scopolamine inflicted neuroinflammation in C57/BL6N mice

Neuroinflammation, one of the major hallmarks in the progression of AD, was frequently reported to facilitate the incidence and expansion of neurodegeneration in the pathology of amnesic brains. Accordingly, we evaluated the modulatory effects of SE-EE on the hippocampal protein expression of inflammatory mediators iNOS and COX-2 using Western blot analyses and inflammatory cytokine levels obtained via ELISA kits with the scopolamine-administered amnesic mice.

#### Effects on inflammatory mediator protein expressions in hippocampus

**Figure 6:**
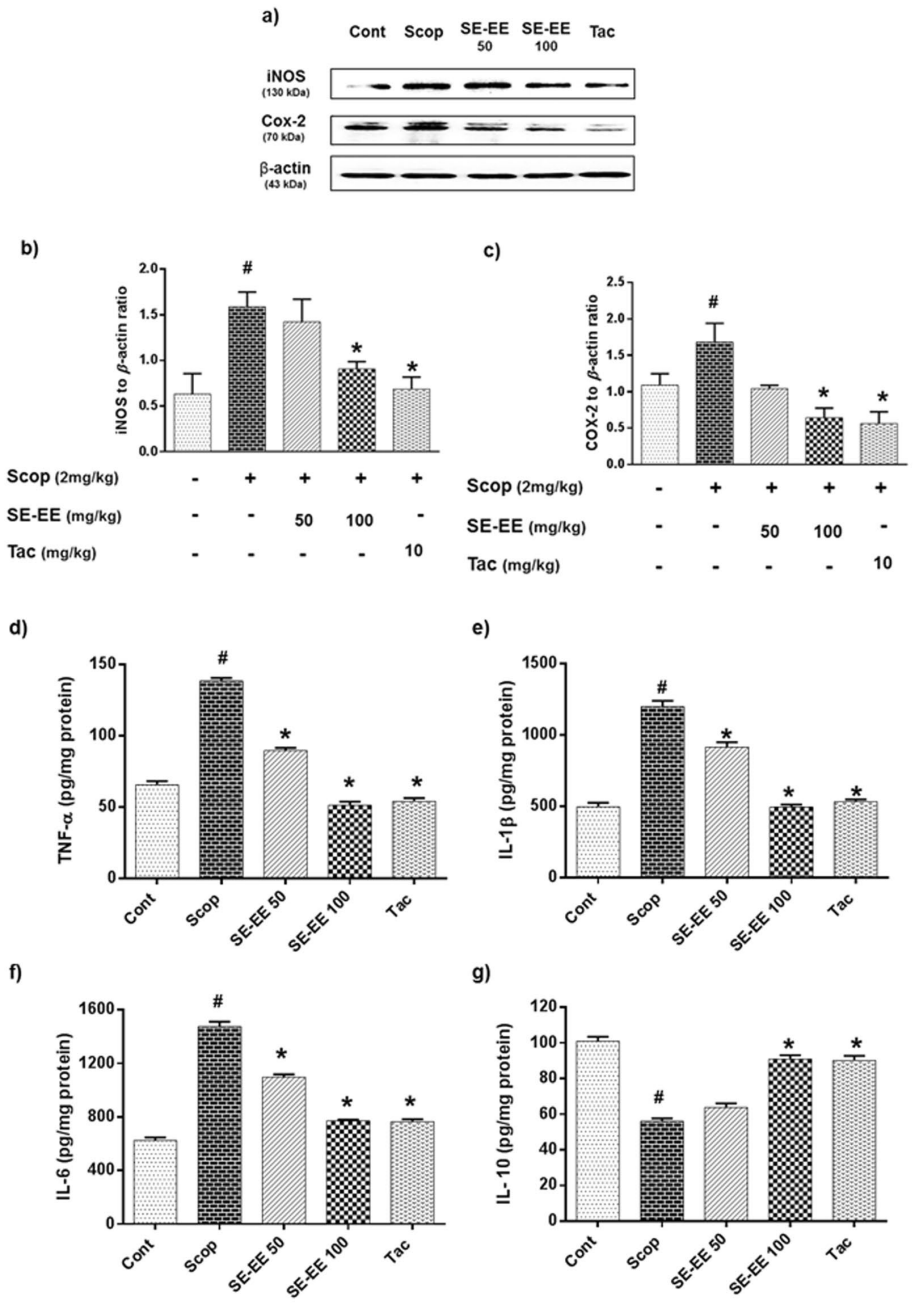
Effects of SE-EE on scopolamine inflicted neuroinflammation in C57/BL6N mice: (**a**) iNOS and COX-2 protein expressions in the hippocampus (n = 3) was determined by western blotting analysis (respective markers were acquired by cropping from the same gel, full length blots are in Supplementary data- S.3.2) and (**b**,**c**) represents the quantification of inflammatory protein expression relative to β-actin, which was achieved using the ImageJ software; (**d**,**e**,**f** and **g**) represent the level of TNF-α, IL-1β, IL-6 and IL-10 proteins in the hippocampus (n = 5, pooled biological replicates), respectively, as acquired via ELISA. Data are expressed as mean ± SD. One-way ANOVA-Tukey’s multiple comparison test was performed. ^#^*p* < 0.05 compared with the control group; *p < 0.05 other treated groups compared with the scopolamine group.

Figure 6a–c shows the results for Western blots of the scopolamine-administered groups, and these exhibited a significant (*p* < 0.05) elevation in the protein expression of iNOS and COX-2, around one-fold higher than control cells. Alternatively, pre-treatment of SE-EE, significantly (*p* < 0.05) suppressed the iNOS and COX-2 expression relatively almost one-fold lesser than scopolamine treated group, and this is found to be comparable to the expression in the tacrine-treated group.

#### Effects on pro- and anti-inflammatory cytokine profiles in the hippocampus

Figure 6d–g show that the scopolamine administration significantly increased the TNF-α, IL-1β, IL-6 (pro-inflammatory cytokines) levels and substantially suppressed the IL-10 (anti-inflammatory cytokine) levels with values of 138.45 ± 2.17 pg/mg protein, 1199 ± 40.93 pg/mg protein, 1475.67 ± 35.10 pg/mg protein and 56.09 ± 1.56 pg/mg protein, respectively, compared to the control group with values of 65.62 ± 2.51 pg/mg protein, 496.67 ± 28.43 pg/mg protein, 624.33 ± 23.59 pg/mg protein and 100.92 ± 2.57 pg/mg protein, respectively. SE-EE administration precisely at 100 mg/kg of b.w. significantly (*p* < 0.05) curbed the levels of TNF-α, IL-1β, IL-6 and elevated the level of IL-10 with the values of 51.37 ± 2.48 pg/mg protein, 494 ± 18.03 pg/mg protein, 771 ± 8.54 pg/mg protein and 90.92 ± 2.17 pg/mg protein, respectively, and is found to be proximal to the tacrine-treated group.

### SE-EE promoted neuronal proliferation and neurogenesis by modulating CREB/BDNF signaling in scopolamine inflicted amnesic C57/BL6N mice

#### Effects on CREB/BDNF protein expression in the hippocampus

**Figure 7:**
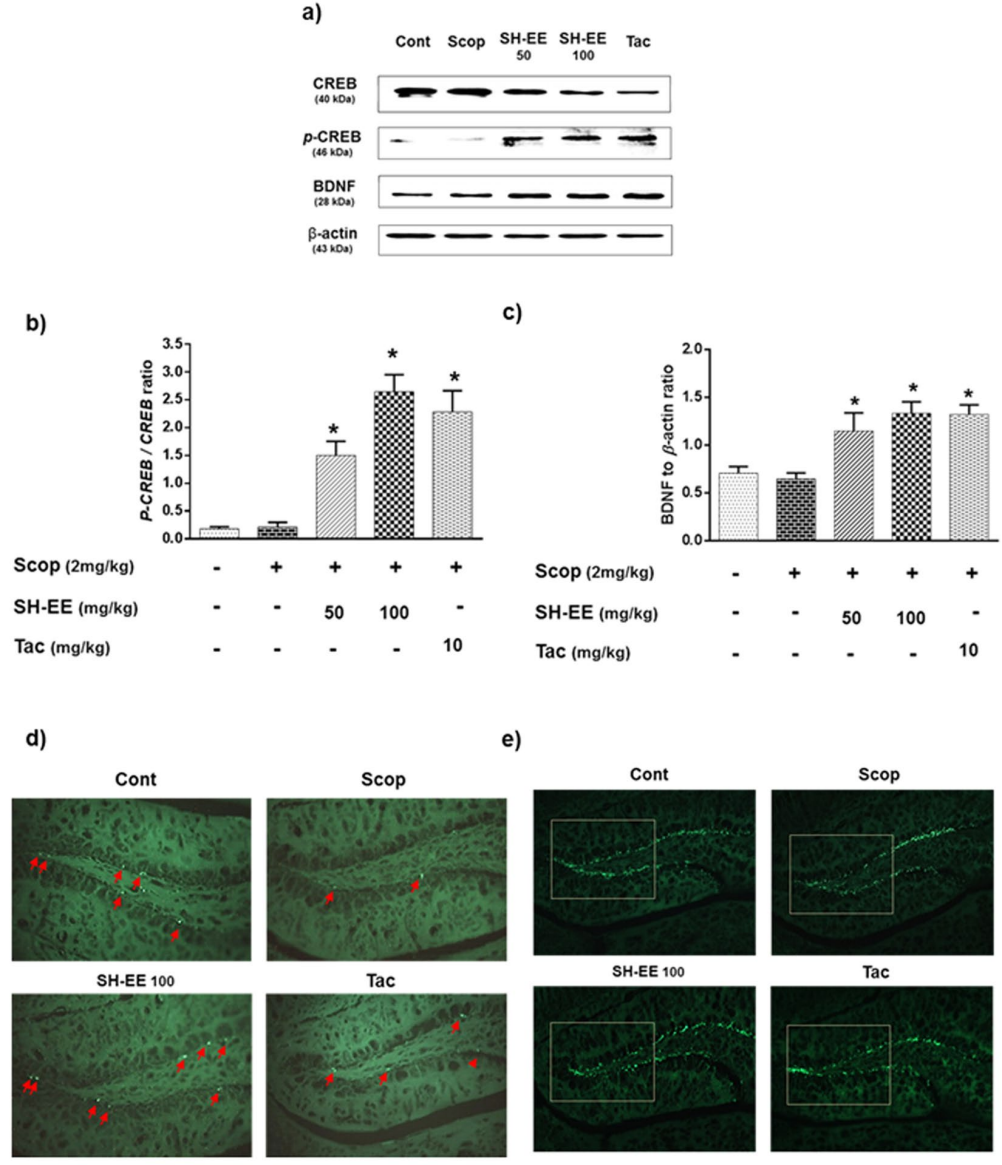
Effects of SE-EE on CREB/BDNF signaling and associated neuronal proliferation and neurogenesis in C57/BL6N mice: (**a**) CREB, *p*-CREB and BDNF protein expressions in the hippocampus (n = 3) were determined via western blotting (respective markers were acquired by cropping from different gels of parallel blotting, full length blots are in Supplementary data- S.3.3) and (**b**,**c**) represent the quantification of protein expression relative to β-actin or phosphorylated proteins using the ImageJ software. The data are expressed as mean ± SD. ^#^*p* < 0.05 compared with the control group; *p < 0.05 other treated groups compared with the scopolamine group; Immunohistochemical analysis of hippocampal-DG regions (n = 3), (**d**) Ki67-positive stained progenitor cell were marked with red arrows in the subgranular zone of DG. (**e**) The DCX-positive stained immature neurons were observed as green flares in the subgranular zone of DG. Representative photomicrographs were taken at magnifications of 200x.

cAMP-response element binding protein (CREB) is a cellular transcription factor that has been reported to play major role in the activation of brain-derived neurotrophic factor (BDNF) gene, which aids in the enrichment of neuronal growth and protection. In accordance, we evaluated the modulatory influence of SE-EE on these neuronal enrichment factors. From Fig. 7a–c, scopolamine-administered mice mildly suppressed the phosphorylated CREB (*p*-CREB) and BDNF expressions. SE-EE administration significantly (*p* < 0.05) increased *p*-CREB and BDNF expressions, in a dose-dependent fashion. At a higher dose, SE-EE (100 mg/kg of b.w.) significantly (*p* < 0.05) increased the *p*-CREB and BDNF by two-fold and one-fold of baseline expressions, respectively, compared to the scopolamine-treated groups. Interestingly the effects of SE-EE were found to be slightly higher than those for the positive control groups.

#### Effects on cell proliferation and neurogenesis in the hippocampus

Subsequently, we evaluated the effects of SE-EE on the neuronal cell proliferation and neurogenesis through the IHC of the hippocampal region using Ki67 and DCX staining respectively. Scopolamine administration substantially inhibited the formation of new granular cells at the hippocampal-Dentate Gyrus (DG) region, which was observed by a decrease in Ki67 stained progenitor cells compared to the control groups (Fig. 7d). Alternatively, pre-treatment with SE-EE attenuated the same through a prominent enrichment of the proliferating cells with apparently higher Ki67 positive stained cells, compared to the scopolamine groups. From the Fig. 7e, the scopolamine injection substantially suppressed the distribution of neuronal bodies and immature neurons in the DG region which was witnessed by irregular DCX staining. SE-EE administration restored adult neurogenesis by enhancing the staining of immature neurons in the DG-subgranular zone (SGZ), compared to the scopolamine inflicted mice, and it was comparable to that of the tacrine-treated groups. The IHC data is consistent with the CREB and BDNF expression, which suggests that SE-EE effectively restored neuronal proliferation and neurogenesis via regulation of CREB/BDNF signaling in scopolamine-administered amnesic mice.

## Discussion

AD is a multifactorial neurodegenerative disorder that inflicts behavioural and cognitive damage in aging adults. Although the etiology of AD is not clear, it was strongly reported to be associated with an interplay in cholinergic impairment, oxidative stress, neuroinflammation and progressive, neuronal loss-mediated neurodegeneration^23,24^. Thus, drug candidates targeting these multiple events can be a potential strategy to curb the progression of AD. In the present study, SE-EE potently inhibited microglia-mediated neuroinflammatory responses in the *in vitro* experiments and attenuated the behavioural-cum-cognitive deficits in scopolamine-induced amnesic mice. Previous reports on the phytoconstituents of SE revealed numerous bioactive secondary metabolites, such as phenolic-flavonoids, poly-saccharides, caffeoylated-quinic acids derivatives, triterpenoid saponins, linolenic acid, scopoletin, pentadecylferulate^25^. Among these, a recent study found that pentadecyl ferulate, a compound of SE, is supposedly responsible for the antioxidant and anti-tumor activity^26^. Another study reported that caffeoyl quinic acid derivatives of SE were potentially responsible for improvements in the high mobility group box 1 protein (HMGB1)-mediated endothelial dysfunction^27^. Recently Won, K. J. *et al*., reported the protective effects of SE extracts and its bioactive fractions against vascular neointima formation in vascular smooth muscle cells. According to the chromatographic data of their study, the extracts of enzyme-digested SE/SE HW bioactive fractions revealed several phenolic-flavonoids (i.e., protocatechuic acid, caffeic acid, p-coumaric acid, ferulic acid, quercetin, isorhamnetin)^28^. Herein, we reported five major compounds, i.e., caffeic acid, trans-ferulic acid, acanthoside B, isorhamnetin, irilin B in SE-EE, which could possibly be responsible for the enhanced bioactivity. To the best of our knowledge we are the first to report on the presence of acanthoside B in SE plant species. Phenolic candidates, such as caffeic acid, trans-ferulic acid and isorhamnetin, are widely-known for their therapeutic properties^29,30^. However, the pharmacological traits of irilin B and acanthoside B have been seldom reported, and thereby future investigations of these candidates shall determine its bioactive potential.

Several scientific evidences suggest that different extraction methods can yield varied phytochemical constituents of the evaluated plant extract and plays critical role in determining its biological potential. In accordance, among the different methodological extracts of SE that were evaluated, the enzyme-digested SE-EE showed substantially higher antioxidant and AChE inhibitory activity *in vitro*. This could possibly attribute to the existence of irilin B and acanthoside B in SE-EE when compare to the chromatographic profiles of the other methodological extracts as reported earlier^28^. Further, SE-EE showed negligible toxicity in rat dopaminergic neural (N27a) cell lines and a considerable toxicity at the evaluated higher dose (500 µg/mL) in human derived neuroblastoma (SE-SY5Y) cell lines (supplementary data S.2.1). Based on this, we conducted *in vitro* investigations of SE-EE on LPS-stimulated microglial cells. Microglial activation is a major event associated with AD-neuroinflammation. At its resting phase, the glial cells may rarely express, inflammatory mediators/cytokines, but upon activation via external toxins (i.e., LPS, scopolamine) or inflammatory stimulus, it produces increased inflammatory mediator proteins - iNOS, COX-2 with a larger release of NO in the brain^31,32^. The results of our study indicated that SE-EE considerably inhibited the excessive NO production and supressed the protein expressions of iNOS, COX-2 in LPS-activated glial cells. Activated microglial cells were also reported to extensively release pro-inflammatory cytokines (TNF-α, IL-1β, IL-6) which influence the further aggravation of diseased conditions^32^. In the present study, SE-EE significantly downregulated the LPS-induced production of TNF-α, IL-1β, IL-6 in BV-2 microglial cells, indicating its potentially beneficial role in suppressing neuroinflammatory-mediated AD progression.

Based on the results of the *in vitro* experiments, we further pursued *in vivo* investigations of SE-EE for their therapeutic potential in scopolamine-induced amnesic AD-like mice. The median doses of 50 and 100 mg/kg of SE-EE were adapted for further *in vivo* studies. Clinically, AD was characterized by behavioural and cognitive impairments, wherein patients tend to gradually lose their memory and are unable to perform their physiological functions^33^. Scopolamine, a competitive muscarinic ACh receptor antagonist, in hand with other molecular events, was reported to actively induce cognitive deficits in mice^34^. Thus, we used scopolamine (2 mg/kg of animal b.w) to induce AD-like cognitive impairment in mice, and we also used Tac (10 mg/kg of animal b.w), an approved AChE inhibitor, as positive control. In this study, scopolamine-induced behavioural and cognitive deficits in mice were evaluated in a Y-maze (consolidation of short/long-term memory and exploratory behaviour) and PAT (memory consolidation and retention) analysis. Pre-administration of SE-EE effectively enhanced the spontaneous alternation of exploratory behaviour and memory retention potential in scopolamine-administered AD mice. In addition, several studies have reported that the central cholinergic system is allied with learning and memory processes, which was regulated via ACh neurotransmission. This was hindered by AChE, a key regulating enzyme of ACh^35,36^. Thus, the inhibition of AChE shall be a potential strategy to curb the cognitive decline in AD patients. Accordingly, several AChE inhibitors were developed and approved for clinical treatment of mild to moderate AD patients^11^. In this study, we found that scopolamine substantially elevated AChE activity in the hippocampus and cortex of the mice brains, which effectively subsided by the pre-treatment of SE-EE. Therefore, our results suggest that SE-EE has the potential to treat cognitive dysfunction in scopolamine-induced AD-like mice, possibly through the inhibition of AChE activity, as reported in earlier studies^37,38^.

Accumulating evidence indicates that the oxidative stress generated by reactive oxygen species (ROS)/reactive nitrogen species (RNS) milieu potentially plays a vital role in the progression of AD in the aging population^39,40^. Typically, the brain demands higher levels of oxygen to carry out its extensive synaptic functions, and it is highly prone to oxidative stress, precisely at the hippocampal and cortex regions^41^. The excessive production of ROS inflicts neurotoxic mechanism by thiol- and lipid- dependent cell membrane peroxidation, suppression of hippocampal plasticity, thereby contributes to the pathogenesis of AD^40,42^. In addition to the behavioural and cognitive decline, scopolamine induced AD mice also evidently mimic the oxidative stress event of AD progression, even though its exact mechanism of oxidative damage is uncertain^43^. In this study, as anticipated, scopolamine administration significantly elevated lipid peroxidation (MDA) levels, indicating the induction of oxidative stress in mice. This alteration was effectively suppressed by SE-EE in both the hippocampus and cerebral cortex, which is consistent with the results of previous reports^44,45^. In general, cellular organisms retain an effective integrated antioxidant defence system comprised of enzymatic and non-enzymatic factors to safeguard tissue from the impact of oxidative stress^46^. In this study, the defence activity of endogenous antioxidant enzymes SOD, CAT and GPx were remarkably suppressed in scopolamine-administered mice. Alternatively, pre-treatment of SE-EE effectively restored the antioxidant defence system by enhancing the activity levels in both the hippocampus and cerebral cortex. Previous reports suggested that phenolic compounds shall have ancillary effects on the amelioration of the antioxidant system, possibly via activation of ARE/Nrf-2 systems. Accordingly, we believe that phenolic enrichment of SE-EE can possibly contribute to its enhanced antioxidant potential^47,48^.

As discussed earlier, neuroinflammation plays a major role in the progression of AD. Thus, we further investigated the anti-neuroinflammatory potential of SE-EE in the AD mice model. Predictably, scopolamine administration upregulated the inflammatory mediators (iNOS and COX-2) and proinflammatory cytokines (TNF-α, IL-1β, IL-6) levels in the hippocampus of mice, and this is in agreement with the results of previous reports^49,50^. However, SE-EE substantially supressed the scopolamine-induced neuroinflammatory mediators/cytokines and also remarkably restored the anti-inflammatory cytokine (IL-10) level in the hippocampus of scopolamine-administered mice. These data can be correlated with the *in vitro* results of our study and thereby support the anti-neuroinflammatory potential of SE-EE.

**Figure 8:**
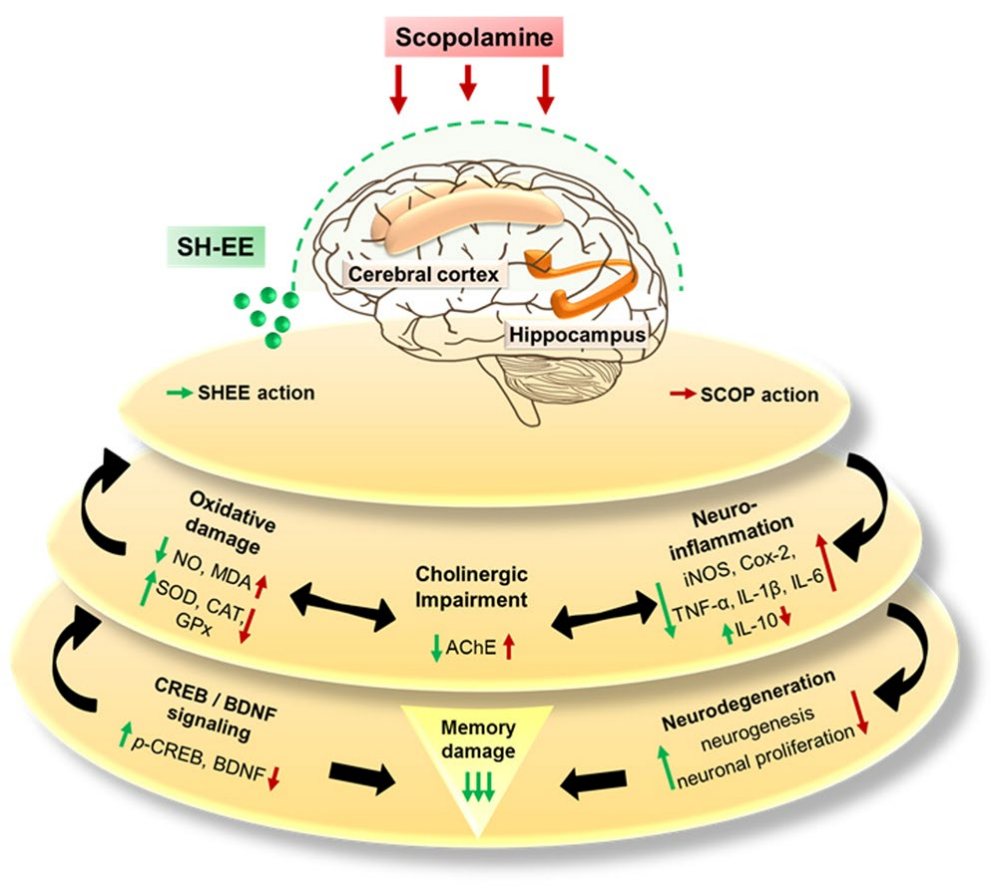
Schematic representation on the ameliorative potential of SE-EE at its molecular mechanistic events associated with scopolamine induced amnesic mice model (the base image of the brain outline in this figure was adapted from pixabay.com).

In adult mammalian brains, neurogenesis occurs through the generation of new neurons as progenitor cells at the sub granular zone of the hippocampal DG region. Accumulating evidence suggests that hippocampal neurogenesis plays vital role in facilitating memory consolidatory functions in the brain^51,52^. Thereby, to enrichment of hippocampal neurogenesis shall be an effective strategy to curb AD progression. In accordance, our results indicated that scopolamine administration substantially reduced the proliferating of neuronal cells and immature neurons in mice hippocampus. This was considerably retrieved in the hippocampal regions of SE-EE administered mice, with enhanced neuronal proliferation and significant expression of budding immature neurons. Subsequently, hippocampal neurogenesis involves several signalling pathways with various transcription factors, of which cyclic AMP (cAMP)-responsive element-binding protein (CREB) is a well-established one^53^. Despite its crucial regulatory role in cell proliferation and neuronal plasticity, CREB was also recently suggested to play a substantial role in learning and memory in adult brains. Phosphorylated CREB (*p*-CREB) facilitates the transcription of several factors, specifically neurotrophins (brain-derived neurotrophic factor, BDNF), which have been reported to potentially aid in memory and cognitive functions^53,54^. In this regard, we extended our molecular investigation towards the impact of SE-EE in the CREB/BDNF signalling pathway. Our study indicated that scopolamine reduced the hippocampal protein expressions of *p*-CREB and BDNF, which was found to be effectively restored in SE-EE pre-treated groups and is comparable to tacrine-treated positive groups. It is also noteworthy that *p-*CREB was reported to possess neuroprotective effects against oxidative stress and also involves the amelioration of cognitive impairment via regulation of the cholinergic system^55,56^. Thus, we suggest that the ameliorative action of SE-EE on oxidative stress and cholinergic impairment might also be substantially achieved through the modulation of CREB/BDNF signalling in scopolamine-induced AD-like mice. The proposed schematic representation of the potential disease modifying mechanisms of SE-EE in scopolamine induced AD-like amnesic mice model was depicted in Fig. 8. In our future work, we intend to identify the potential compounds responsible for the enhanced therapeutic activity of SE-EE and also work on the bioavailability profiles of SE-EE/bioactive candidates in blood and brain to determine its *in vivo* PK/PD profile. This shall eventually aid to extrapolate a suitable dosage regimen for further translational clinical research to treat progressive AD-related complications.

## Materials and Methods

### Reagents

Lipopolysaccharide (LPS), scopolamine hydrochloride (SCOP), tacrine hydrochloride hydrate (TAC) and 3-(4,5-dimethylthiazol-2-yl)-2,5-diphenyltetrazolium bromide (MTT) were purchased from Sigma-Aldrich (St. Louis, MO, USA). RIPA buffer (10×) was acquired from Millipore (Milford, MA, USA). Protease and phosphatase inhibitor cocktail tablets were obtained from Roche (Indianapolis, IN, USA). Tween 80 was acquired from Calbiochem (Gibbstown, NJ, USA). Dulbecco’s modified Eagle’s medium (DMEM) and fetal bovine serum (FBS) were obtained from Gibco-BRL Technologies (Carlsbad, CA, USA). All other chemicals were of analytical grade and were obtained from Sigma, unless otherwise mentioned.

### Plant materials and extraction procedure

The stem and leaves of *Salicornia europaea L*. (SE) were collected from the western seashore marshes of South Korea and were registered as voucher specimens deposited at the R&D center of Phyto Corporation (Seoul National University, South Korea). The SE-EE extraction procedure (Fig. 1a) was described briefly in Supplementary Section S.1.1. Although the residual extract that was obtained is commercially known as enzyme-digested PhytoMeal ethanol extract (PM-EE) by Phyto Corporation, we refer to it as enzyme-digested desalted *Salicornia europaea* ethanol extract (SE-EE) throughout the study for reader clarity.

### Chromatographic and phytochemical profile of SE-EE

To analyze the major low-molecular-weight compounds in SE-EE, 20 mg of SE-EE were dissolved in methanol and subjected to a chromatographic analysis. A brief description of the HPLC conditions and associated procedures are mentioned in Supplementary Section S.1.2. A brief description of the experiments regarding the total phenolic content and total flavonoid content of SE-EE is provided in Supplementary Sections S.1.3 and S.1.4, respectively.

### *In vitro* antioxidant and anti-acetylcholinesterase potential of SE-EE

A brief description of the *in vitro* DPPH radical scavenging and AChE activity is provided in Supplementary Section S.1.5.

### Cell culture and treatment

BV-2 microglial cells were obtained and cultured as previously described^57^. Briefly, the BV-2 cells were cultured and maintained in DMEM supplemented with 5% FBS and 50 μg/mL penicillin-streptomycin in a 37 °C humidified incubator supplied with 5% CO_2_ and 95% O_2_. The microglial cells were seeded at a density of 5 × 10^4^ cells/mL and were pretreated for 1 h with various concentrations of SE-EE (20, 100 and 200 μg/mL) and were subsequently incubated with LPS (200 ng/mL) for the indicated time points (6 and 24 h).

### Animals and treatment

Male C57BL/6 N mice strains (n = 8 per group; age, 8–9 weeks; weight, 24–27 g) were obtained from Deahan Bio Link (Eumseong, Korea). All animals were housed in a controlled environment (23 ± 1 °C; 50% ± 5% humidity; 12 h dark-light cycle) and allowed water and food *ad libitum*. After a week of acclimatization, the animals were randomly divided into experimental groups. The design of the animal experiments is shown in Fig. 4a. In brief, the animals were divided into five groups: Control group (n = 8; 0.9% saline, *i.p*.), Scop 2 mg/kg, (n = 8; Scop in 0.9% saline, *i.p*.), SE-EE_50_ (n = 8; SE-EE 50 mg/kg, *p.o*. + Scop 2 mg/kg *i.p*.), SE-EE_100_ (n = 8; SE-EE 100 mg/kg, *p.o*. + Scop 2 mg/kg *i.p*.) and Tac (n = 8; Tac 10 mg/kg, *p.o*. + Scop 2 mg/kg *i.p*.). SE-EE and tacrine were administered using an oral gavage dissolved in 0.9% saline containing 1% Tween 80 and 0.9% saline, respectively, for 7 days prior to Scop injection. All experiments were accomplished in accordance with the Principles of Laboratory Animal Care (NIH publication no. 85–23, revised 1985) and were approved by the Konkuk University Institutional Animal Care and Use Committee (KU17068).

### Cell viability and NO assays

BV-2 cells were seeded at a density of 5 × 10^4^ cells/mL and exposed to LPS (200 ng/mL), followed by 1 h pretreatment of SE-EE at the indicated concentrations (20, 100 and 200 μg/mL) for 24 h. Subsequently, MTT (0.5 mg/ml) was added to each well and incubated for 4 h at 37 °C with 5% CO_2_ and 95% O_2_. The supernatants were carefully discarded from each well, and DMSO was added to dissolve the formazan crystals in viable cells. The plates were read at 540 nm absorbance using a microplate reader (Tecan Trading AG, Switzerland). The NO assay was performed as previously described^57^. In brief, microglial cells (5 × 10^4^ cells/mL) were exposed to LPS (200 ng/mL), with or without pretreatment of SE-EE (20, 100 and 200 μg/mL) for 24 h. The supernatants were carefully collected from each well and were subjected to an NO release assay using commercial Griess reagent (1 vol. 0.1% naphthylethylenediamine and 1 vol. 1% sulfanilamide in 5% H3PO4). The colorimetric changes were acquired at 540 nm using a microplate reader.

### Total RNA isolation and reverse transcription polymerase chain reaction (RT-PCR)

The BV-2 cells (5 × 10^4^ cells/mL) exposed to LPS (200 ng/mL), with or without pretreatment of SE-EE (20, 100 and 200 μg/mL) for 6 h, were adopted for RNA studies. The total RNA from the treated cells was acquired using Trizol reagent (Invitrogen Life Technologies, CA, USA) according to the manufacturer’s instructions. The cDNA synthesis was attained by reverse transcribing the RNA (2.5 μg) using GoScriptTM Reverse Transcription System (Promega, USA), according to the manufacturer’s instructions. Subsequently, PCR amplification was achieved using precise primers, as described earlier, analyzed in 1% agarose gels. For quantification, the gels were photographed and the pixel intensity for each band was determined using the ImageJ (NIH) software and was normalized to the band intensity of GAPDH mRNA. The results are representative of three independent experiments.

### Immunoblot analysis

The BV-2 cells (5 × 10^4^ cells/mL) exposed to LPS (200 ng/mL), with or without pretreatment of SE-EE (20, 100 and 200 μg/mL) for 18 h, were adopted in immunoblot studies. After treatment, the cells were washed twice with PBS and lysed for 10 min at 4 °C using 1 × RIPA lysis buffer (with protease and phosphatase inhibitor). The cell lysates were centrifuged at 14,000 rpm, 4 °C, and the supernatants were collected for further investigation. The protein concentration of each sample was obtained and normalized using a DC Protein Assay kit (Bio-Rad). Equal amounts of proteins (20–40 μg) were loaded and separated electrophoretically in 10% sodium dodecyl sulfate-polyacrylamide gel and were transferred to polyvinylidenedifluoride membranes (Millipore, Bedford, MA, USA). The membranes were then incubated overnight at 4 °C, with corresponding primary antibodies to anti-inducible nitric oxide synthase and anti-cyclooxygenase-2 followed by incubation with horseradish peroxidase-conjugated specific secondary antibodies (1:2000; Cell signaling, MA), for 1 h. The blots were visualized using a PowerOpti-ECL kit obtained from Animal Genetics Inc. (Gyeonggi-do, Korea) as part of the detection system, following the recommended procedures.

### Behavioral studies

#### Step-through passive avoidance test (PAT)

A brief description of the experimentation for the step-through passive avoidance test was mentioned in Supplementary Section S.1.6.

#### Spontaneous alternation performance (Y-maze test)

A brief description of the spontaneous alternation test is mentioned in Supplementary Section S.1.7.

### Tissue acquisition and protein quantification

All mice were anesthetized and sacrificed after 1 hour following the y-maze. The whole brain was carefully excised from the skull, washed twice using cold saline solution, followed by acquisition of the hippocampus and cerebral cortex, as previously described^34^. A detailed description of the tissue acquisition and protein quantification were mentioned in Supplementary Section S.1.8.

### Biochemical analyses

#### AChE activity

AChE activity in the hippocampus and cerebral cortex homogenates was determined using a Biochain kit (Biochain, CA), according to the manufacturer’s protocol as previously described^34^. The absorbance was measured at 410 nm using a UV spectrophotometer.

#### Lipid peroxidation

The level of lipid peroxidation activity in the hippocampus and cerebral cortex homogenates was determined by measuring the MDA levels using a lipid peroxidation (MDA) colorimetric/fluorometric assay kit (BioVision, USA, CA) according to the manufacturer’s protocol, as previously described^58^. The absorbance was measured at 410 nm using a UV spectrophotometer.

#### SOD, CAT and GPx analysis

SOD, CAT and GPx activity of the tissue homogenates were determined using Oxiselect superoxide dismutase, catalase assay kits (Cell Biolabs, Dan Diego, CA) and Glutathione peroxidase activity colorimetric assay kit (BioVision, USA, CA) respectively, according to the manufacturer’s protocol, as previously described^58,59^. The absorbance of SOD, CAT and GPx activities were measured at 490 nm, 520 nm and 340 nm respectively, using a UV spectrophotometer.

### ELISA

The level of pro-inflammatory (TNF-α, IL-1β, IL-6) and anti-inflammatory (IL-10) cytokines in the hippocampus were measured using commercially available ELISA kits (R&D systems, Minneapolis, MN) according to the manufacturer’s protocol, as previously described^34^.

### Western blot analysis

The expressions of iNOS, COX-2, CREB/phospho-CREB and BDNF proteins in the hippocampus was determined using a Western blot, as previously described^34^. The Western blot experiments are briefly described in Supplementary Section S.1.9.

### Immunohistochemical (IHC) stain analysis

The neuronal cell proliferation and neurogenesis in the hippocampus was evaluated using Ki67 and DCX staining, respectively, via IHC analysis, as previously described^60^, with slight modifications. The experimental protocol for tissue processing, sectioning, staining and IHC analysis was briefly described in Supplementary Section S.1.10.

### Statistical analysis

All data were analyzed using Graph Pad Prism, version 5.01 (La Jolla, CA, USA). These were expressed as mean ± SD analyzed using a One-Way ANOVA followed by Tukey’s Multiple Comparison Test. In the present study, both the *in vitro* and *in vivo* analysis were carried out by comparing a control group to a negative control group and then a negative control with the treated and/or positive control groups. In all cases, the probability values of *p* < 0.05 were considered to be statistically significant.

## Conclusion

In summary, we have demonstrated that SE-EE possess anti-amnesic effects and regulates behavioral-cum-cognitive deficits in scopolamine-induced AD-like mice, possibly by enhancing hippocampal neurogenesis through the regulation of CREB/BDNF signaling. In addition, anti-cholinesterase, antioxidative and anti-neuroinflammatory potential of SE-EE facilitates the curbing of multifactorial events associated with neurodegenerative complications. Overall, our study suggests that SE-EE can be developed as an effective therapeutic/dietary supplement to treat amnesic neurodegenerative complications. However, further PK/PD studies of SE-EE/bioactive candidates can pave way to determine the bioavailability and dosage regimen for future translational research.

## Electronic supplementary material

Supplementary Information
