# Supplementary material for: Ameliorative potential of desalted Salicornia europaea L. extract in multifaceted Alzheimer’s-like scopolamine-induced amnesic mice model
Source: Sci Rep. 2018 May 8;8:7174. doi: 10.1038/s41598-018-25381-0 (PMC5940894; doi:10.1038/s41598-018-25381-0)
Supplement: Supplementary file 1 — Supplementary Information [file 41598_2018_25381_MOESM1_ESM.docx]

**Ameliorative potential of desalted *Salicornia europaea L.* extract in multifaceted Alzheimer’s-like scopolamine-induced amnesic mice model.**

Govindarajan Karthivashan ^a^, Shin-Young Park ^b^, Mee-Hyang Kweon ^d^, Joon Soo Kim ^b^, Md. Ezazul Haque ^b^, Duk-Yeon Cho ^b^, In-Su Kim ^a^, Eun-Ah Cho ^d^, Palanivel Ganesan ^a,c^ and Dong-Kug Choi * ^a,b^

*^a^* *Department of Biotechnology, College of Biomedical and Health Science, Research Institute of Inflammatory Diseases Konkuk University, Chungju 27478, Republic of Korea. ^b^ Department of Applied Life Science, Graduate school of Konkuk University, Chungju 27478, Republic of Korea. ^c^ Nanotechnology research center College of Biomedical and Health Science, Konkuk University, Chungju 27478, Republic of Korea. ^d^ Research center, Phyto corporation, Seoul 08826, Republic of Korea.* * *(Tel) +82-43-840-3610; (Fax) +82-43-840-3872; (E-mail)* [*choidk@kku.ac.kr*](mailto:choidk@kku.ac.kr)

**Supplementary sections:**

**S.1. Materials and methods**

*S.1.1 Preparation of SE-EE extracts*

The fresh aerial parts (50 kg) of the plant were washed thoroughly, freeze-dried using lyophilizer (FDU-2200, EYELA, Japan), powdered using a blender (EBR9804S, Electrolux, Stockholm, Sweden) and stored in an airtight container for further process. Primarily, the powdered SE was desalted by cold-water extraction procedure as described previously^60^. The obtained desalted SE powder or also known as PhytoMeal (PM) (500 g), was suspended in distilled water containing 1% pectinase/cellulose (Connell Bros, Croydon South Vic, Australia) and incubated at 50 °C for 15 h for enzyme digestion. Further, the enzyme digested SE-E/PM-E was subjected to ethanol (60 %) reflux for 3 h, twice followed by filtration. The filtrate was condensed using a rotary evaporator and the residual SE-EE/PM-EE was freeze-dried and stored at -20 °C for further analysis.

*S.1.2 HPLC conditions*

HPLC experiments were performed using an Agilent HPLC instrument (Infinity 1260, USA) with a 1260 quaternary pump, 1260 ALS auto sampler, 1260 DAD diode-array detector, and 1260 TCC thermo-statted column compartment. The SE-EE sample (20 mg/mL, 5 μL) was injected into a Zorbax Eclipse Plus C18 analytical column (4.6 × 150 mm, 3.5 μm, Agilent, USA) and analyzed with a gradient eluent of acetonitrile and water, at a flow rate of 1.0 mL/min and a column temperature of 25 °C. HPLC profiles and the UV spectrum were recorded using an Agilent UV detector (1260 DAD, 190–400 nm, 20-nm step) at 300 nm/reference 360 nm. To identify the major compounds, SE-EE dissolved in methanol was ionized by spray ionization (150~ 2000m/z) and its ESI-MS spectra was obtained using a LC-ESI mass spectrometer (Thermo Finnigan LTQ, X-caliber software, USA) with both positive and negative modes. Subsequently, by comparing the HPLC retention time and the UV absorption λ-maxima of the authentic compounds (Sigma Co, USA), several major compounds of SE-EE were identified and confirmed. For quantitative analysis of the identified major compounds, standard solutions (1.0 mg/mL in methanol) of individual authentic compound were diluted to the required concentrations (50, 20, 10, 5, 2, 1 μg/mL) for obtaining respective calibration curves. They were injected onto HPLC system to generate calibration functions, which were calculated using peak area (y axis) and concentration (x axis). Five μL of SE-EE sample (20 mg/mL) was loaded onto HPLC system under the same condition.

*S.1.3 Total Phenolic content*

The total carbohydrate and uronic acid content in SE-EE was assessed by the phenol-sulphuric method^61^ and p-hydroxy diphenyl method^62^, respectively. The protein content in SE-EE was measured by the method of Lowry et al.,^63^.

Total Phenolic content was determined using Folin-Ciocalteau reagents^64^. Briefly, SE-EE or gallic acid standard (20 μL) were mixed with 250 μL of 2% sodium bicarbonate and incubated at room temperature for 5 min, and then 16 μL of 50% Folin-Ciocalteu reagent (prediluted 2-fold with distilled water) was added to the mixture. After incubating for another 30 min at room temperature, the absorbance was measured at 725 nm. Aqueous solutions of known gallic acid concentrations in the range of 10 – 500 μg /mL were used for calibration. Results were expressed as mg gallic acid equivalents (GAE)/g sample.

*S.1.4 Total flavonoid content*

Total flavonoids content was measured using Abdel-Hameed method^65^. Briefly, SE-EE or rutin standard dissolved in methanol (20 μL) were mixed with 200 μL of 2% diethylene glycol and allowed to incubate at 30˚C for 60min, and then 6 μL of 1N NaOH solution was added to the mixture. After 10 min the absorbance was measured at 420 nm. Methanolic solutions of known rutin concentrations in the range of 10 – 1000 μg /mL were used for calibration and the results were expressed as mg rutin equivalents (REQ)/ g sample.

*S.1.5 In vitro antioxidant and anti-cholinesterase potential of SE-EE*

The *in vitro* antioxidant activity of SE-EE was determined using DPPH radical scavenging assay based on the scavenging activity of stable DPPH free radicals^66^. Reaction mixtures containing SE-EE dissolved in methanol and 200μM DPPH in a 96-well microtiter plate were incubated at 37 °C for 30 min. Subsequently the absorbance was measured at 520 nm, and percentage of inhibition was calculated. The *in vitro* AChE activity of SE-EE was measured by the spectrophotometric method developed by Ellman et al.,^67^, with slight modifications, having acetylcholine iodide as substrates respectively. The rate of thiocholine production is determined by the continuous reaction of the thiol with 5,5-dithiobis-2-nitrobenzoate (DTNB) ion to produce the yellow anion of 5-thio-2-nitrobenzoic acid. The absorbance of the mixture was read at 412 nm at intervals of 30 s for 5 min immediately followed by the addition of substrate and the percentage of inhibition was calculated.

*S.1.6 Step-through passive avoidance test (PAT):*

PAT experiment was conducted using Gemini active and passive avoidance instrument (San Diego Instruments, San Diego, CA) allied with a computerized system as described earlier^24^, with slight modification. In brief, individual animals were acclimatized with the PAT instrument for 2-3 mins before acquisition tasking without shock treatment (0.5mA). On the day of acquisition, individual animal was accustomed in the lighted compartment for 30 s, followed by computer controlled opening of guillotine door, and the animal was subjected to a trial of 270 s. On the entry of animal to the dark compartment, the door was programmed to shut and the animal was punished with single low intensity foot shock of 0.5 mA for 5 s, followed by recording of time latencies (LT). Subsequently after 24 h, retention tasking was performed, with the same procedure, except no shock punishment was delivered to the animals when enters into dark compartment. The criterion for learning was taken as an increase in the LT on retention trial as compared to acquisition trial.

*S.1.7 Spontaneous alternation performance (Y-maze test):*

Spontaneous alternation behavior was evaluated to determine the instant memory functioning and exploratory behavior of animals using y-maze test as described previously^24^, with slight modifications. In brief, each animal was introduced naive at the one end of the “Y” shaped maze groove and allowed to explore freely throughout the maze for 8 min. Total number of arm entries (i.e., hind paw of the animal was completely placed inside the arm) by the animals were documented visually by a person blinded to the experimental groups. Alternation was determined based on the consecutive entries of animals into the three arms, on overlapping triplet sets. The percentage of alternation can be calculated using the following formula:

Percentage alternation = [(number of alternations) / (total number of arm entries -2)] x 100

*S.1.8 Tissue acquisition and protein quantification:*

In each group, three mouse brains were fixed in 4% paraformaldehyde via cardiac perfusion and the hippocampi and/or cerebral cortex of remaining five mice was used for biochemical, western blot and ELISA analysis. A portion of the tissue sample was homogenized using RIPA lysis buffer (Millipore) with protease inhibitor cocktail (Roche, Mannheim, Germany) and the supernatants were stored at -70^◦^C for western blot analysis and the remaining portion was used for biochemical and ELISA analysis. The protein level of the homogenates has been quantified using Bio-Rad DC Protein Assay kit according to the manufacturer’s protocol and normalized for further analysis.

*S.1.9 Western blot analysis:*

In brief, the proteins from the homogenates were separated in 10% polyacrylamide gels and transferred to PVDF membrane (Millipore, Bedford, MA, USA). After blocking the membranes with 5% BSA, the membranes were incubated overnight at 4°C with primary antibodies: anti-iNOS (1:2000), anti-COX-2 (1:2000), anti-CREB (1:1000), anti-*p-*CREB, (1:1000), anti-BDNF (1:1000) and anti-β-actin (1:1000). The membranes were washed and incubated with respective HRP-conjugated secondary antibodies (1:10000). The blots were visualized using by a Davinch-Chemi & Fluoro Imaging System (Seoul, Korea) and their relative band densities were analyzed by ImageJ software (version-1.47).

*S.1.10 Immunohistochemical (IHC) stain sectioning and analysis*

The brain tissues immersed in the fixative solution for 4 h, was subjected to 30% sucrose solution and cryoprotected by embedding in tissue-freezing medium, and were sliced into coronal sections (20 µm) using cryostat. The free-floating sections were exposed to 1% H_2_O_2_ in PBS for quenching of endogenous peroxide for 15 mins, followed by rocking in blocking buffer (normal serum in 0.1M PBS and 0.3% Triton X-100) for 1 h with gentle agitation. Subsequently the sections were washed and incubated with primary Ki67 (1:200), DCX (1:200) antibodies overnight at 4^◦^C respectively. After washing with 0.1M PBS/ 0.3% Triton X-100, the tissue sections were incubated with Alexa Fluor 488-conjugated donkey anti-rabbit (A-21206) -1:1000 (Invitrogen, Carlsbad, CA, USA) for 2h at RT, with gentle agitation. All the immunoreacted images were captured using a fluorescence microscope (Carl Zeiss Inc., Germany).

**S.2. Supplementary data**

*S.2.1 SE-EE cytotoxicity data in neuroblastoma (SH-SY5Y) and rat dopaminergic neural (N27a) cell lines:*


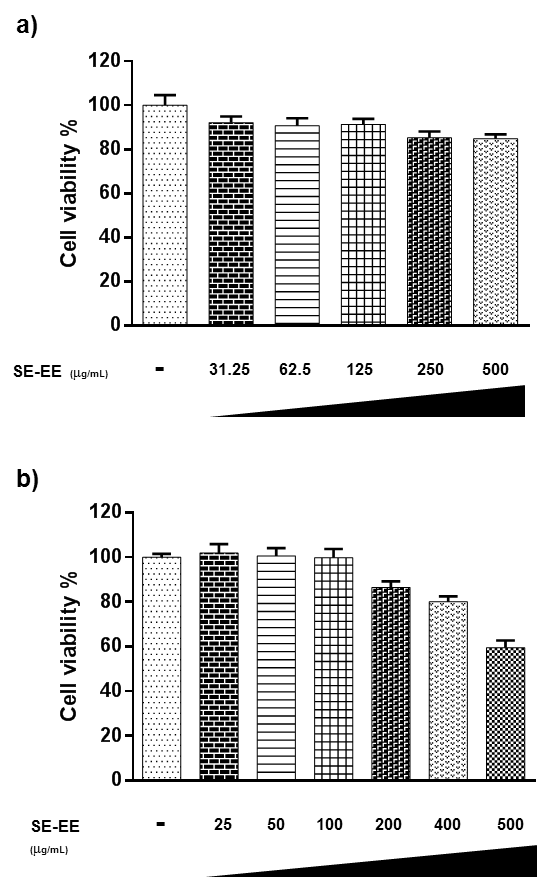


**S.2.1. Cytotoxicity profile of SE-EE in dopaminergic and neuroblastoma cell lines :** The a) N27a dopaminergic neural cells and b) SH-SY5Y neuroblastoma cells were incubated with the described concentrations of SE-EE for 24 h, followed by measuring of cell viability percentage through MTT assay. SE-EE does not exhibit substantial toxicity in the dopaminergic cells at the evaluated doses however at the evaluated higher dose of 500 µg/ml, SE-EE shows toxicity in neuroblastoma cell lines. Data are expressed as mean ± SD of three independent experiments. One-way ANOVA-Tukey’s multiple comparison test was performed where, # *p*<0.05 LPS treated cells compared with the control cells.

**S.3. Supplementary data - full length gels and blots**

*S.3.1 Effects of SE-EE on LPS induced iNOS and COX-2 expressions in BV-2 microglial cells:
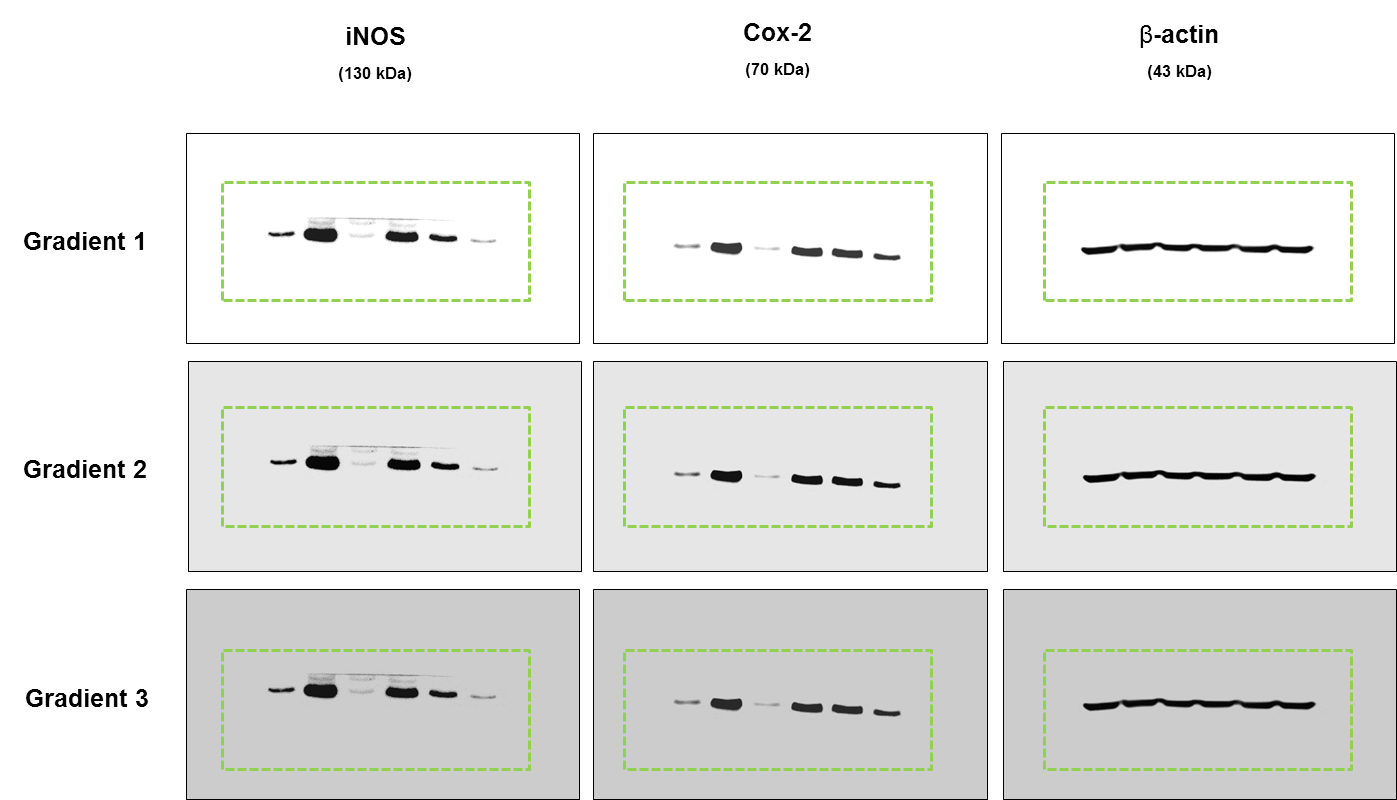
*

*S.3.2 Effects of SE-EE on Scopolamine induced iNOS and COX-2 expressions in C57BL/6N mice:*

*
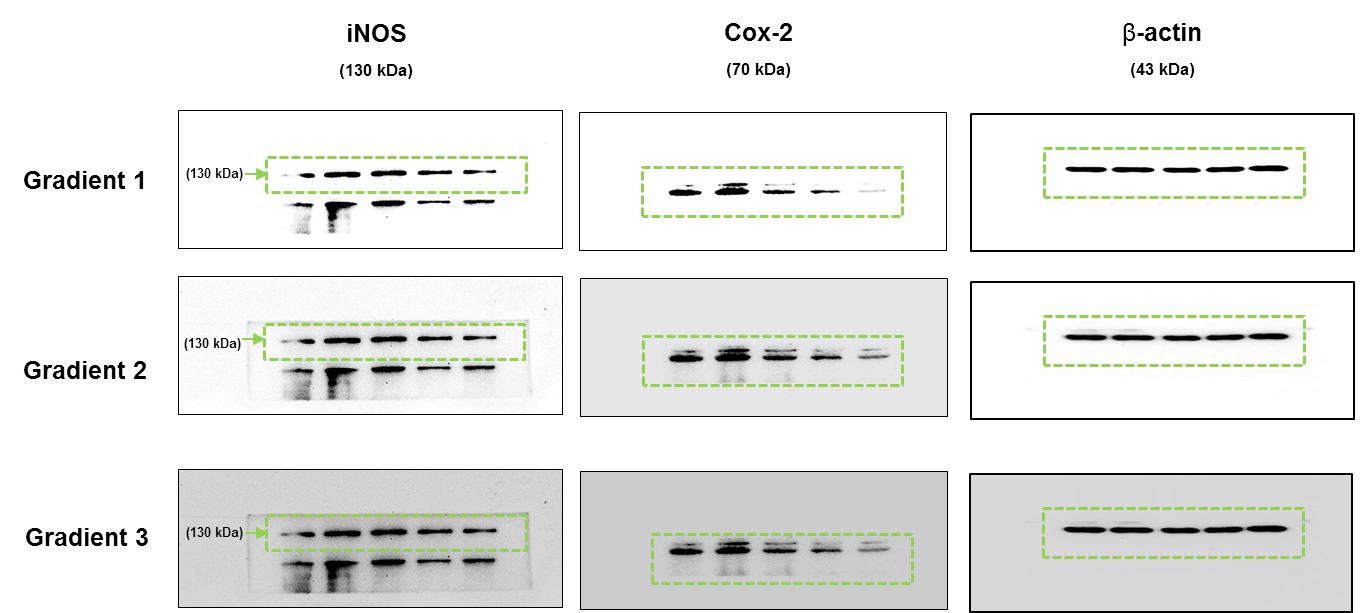
*

*S.3.3 Effects of SE-EE on Scopolamine induced CREB, p-CREB and BDNF expressions in C57BL/6N mice:*


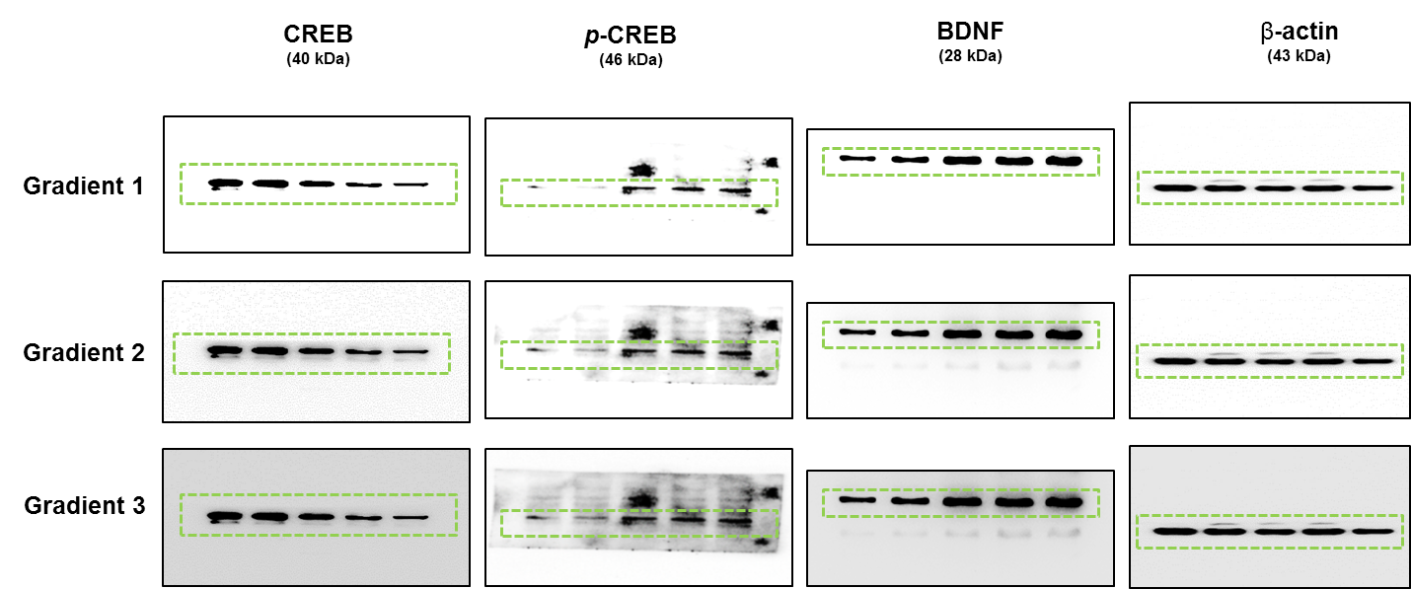


References

61.

Filisetti-Cozzi, T. M. & Carpita, N. C. Measurement of uronic acids without interference from neutral sugars. *Analytical biochemistry* **197**, 157–162 (1991).

62.

DuBois, M., Gilles, K. A., Hamilton, J. K. & Rebers, P. t. & Smith, F. Colorimetric method for determination of sugars and related substances. *Analytical chemistry* **28**, 350–356 (1956).

63.

Lowry, O. H., Rosebrough, N. J., Farr, A. L. & Randall, R. J. Protein measurement with the Folin phenol reagent. *The Journal of biological chemistry* **193**, 265–275 (1951).

64.

Singleton, V. L. & Rossi, J. A. Colorimetry of Total Phenolics with Phosphomolybdic-Phosphotungstic Acid Reagents. *American Journal of Enology and Viticulture***16**, 144 (1965).

65.

Abdel-Hameed, E.-S. S., Bazaid, S. A. & Salman, M. S. Characterization of the Phytochemical Constituents of Taif Rose and Its Antioxidant and Anticancer Activities. *BioMed research international* **2013**, 13, https://doi.org/10.1155/2013/345465 (2013).

66.

Chen, Y., Wang, M., Rosen, R. T. & Ho, C. T. 2,2-Diphenyl-1-picrylhydrazyl radical-scavenging active components from Polygonum multiflorum thunb. *Journal of agricultural and food chemistry* **47**, 2226–2228 (1999).

67.

Ellman, G. L., Courtney, K. D., Andres, V. Jr & Feather-Stone, R. M. A new and rapid colorimetric determination of acetylcholinesterase activity. *Biochem Pharmacol***7**, 88–95 (1961).
